# A Mendelian randomization analysis of inflammatory skin disease risk due to mineral deficiencies

**DOI:** 10.3389/fnut.2024.1404117

**Published:** 2024-10-14

**Authors:** Ronghui Wu, Hao Tian, Tianqi Zhao, Yangyang Tian, Xianhua Jin, Mingji Zhu

**Affiliations:** ^1^Department of Dermatology, China-Japan Union Hospital of Jilin University, Changchun, China; ^2^Department of Dermatology, Chinese People's Liberation Army General Hospital, Beijing, China; ^3^Department of Orthopedics, China-Japan Union Hospital of Jilin University, Changchun, China

**Keywords:** mineral, iron metabolism, Mendelian randomization, psoriasis, atopic dermatitis, acne vulgaris

## Abstract

**Background:**

Mineral deficiencies, such as iron (Fe), zinc (Zn), and selenium (Se), play crucial roles in inflammation and immune responses and are linked to chronic inflammatory skin diseases. This study used genome-wide association study (GWAS) data and Mendelian randomization (MR) to investigate the genetic causality among serum levels of five minerals (Fe, Cu, Zn, Se, Ca), three iron metabolism indicators (TSAT, TIBC, ferritin), and three chronic inflammatory skin diseases [psoriasis (PS), atopic dermatitis (AD), acne vulgaris (AV)].

**Methods:**

Two-sample MR analyses using the “TwoSample MR” package in R were conducted with aggregate outcome data from the FinnGen database. The inverse-variance-weighted (IVW) method was applied to assess causal relationships between mineral levels and disease outcomes. Robustness was examined via heterogeneity and pleiotropy tests.

**Results:**

IVW analysis showed significant association between blood transferrin saturation (TSAT) and PS (*p* = 0.004, OR = 1.18). Serum Zn and Se levels showed inverse correlation with AD (*p* = 0.039, OR = 0.92). However, due to limited SNPs, robustness was reduced.

**Conclusion:**

TSAT is genetically linked to PS, highlighting iron homeostasis in disease development. Zn and Se intake may reduce AD risk.

## 1 Introduction

Chronic inflammatory skin disease, including psoriasis (PS), atopic dermatitis (AD), and acne vulgaris (AV), significantly compromise a patient's wellbeing, creating psychological and financial distress. This is attributable to their recurrent nature, the challenging treatment landscape, their intricate etiological mechanisms, and their propensity to produce complications, such as persistent pruritus, scarring, and hyperpigmentation (1–3). Importantly, stimulation of inflammation activates signaling pathways such as STAT3 and NF-κB thereby promoting the proliferation and metastasis of tumor cells (4). Chronic inflammation has been extensively validated and confirmed to increase the risk of malignant skin tumors and cancer (5). In a meta-analysis investigating the risk of malignancies in PS patients, it was found that the incidence of cancer is elevated among PS patients [standardized incidence ratio, 1.16 (95% CI, 1.07–1.25)] (6). Additionally, an increased risk of squamous cell carcinoma and basal cell carcinoma was observed in a cohort of AD patients (7). Consequently, chronic inflammatory skin diseases not only impair patients' quality of life but also pose a threat to their mortality. Identifying key therapeutic targets for chronic inflammatory skin diseases can serve not only in disease prevention but also in the prevention of cutaneous malignancies.

Recent clinical investigations and meta-analytical reviews have revealed a compelling correlation between serum mineral concentrations and the pathogenesis of chronic inflammatory dermatological conditions. For example, with 5% zinc (Zn) present in the epidermis, it facilitates wound healing and exhibits anti-inflammatory effects (8). Selenium (Se) proteins, such as glutathione peroxidase (GPx) and thioredoxin reductase (TrxR) protein family, are involved in antioxidant defense and redox state regulation (9). However, the effect of dietary mineral intake on chronic inflammatory skin diseases remains under debate. For example, Leveque et al. (10) revealed that the average iron (Fe) levels in individuals with PS were markedly elevated compared to those in the control cohort not suffering from PS. Conversely, Chen et al. (11), through meta-analytical scrutiny, deduced that Fe concentrations did not significantly differ between patients with PS and the control group. Ingestion of minerals such as Zn and Se has been effectively associated with the prevention and amelioration of AD (12), and supplementation with trace elements, including Zn and copper (Cu), has been found to substantially diminish the prevalence of AV (13). Consequently, an enhanced understanding of the causal dynamics between mineral elements and chronic inflammatory dermatoses will facilitate disease prophylaxis and identify potential targets for effective lifestyle and pharmacological interventions.

Following the widespread dissemination of data from genome-wide association studies (GWAS) (14), complemented by significant contributions from FinnGen, the UK Biobank (UKB), and the European Association for Gray Literature Exploitation (EAGLE), the statistical methodology known as Mendelian randomization (MR) (15), which leverages genetic variations as instrumental variables (IV) to clarify causal links between exposure factors and outcomes, has gained widespread acceptance within the medical community. Because of its foundation in the principle of random allocation of single nucleotide polymorphisms (SNPs) (16), MR is impervious to confounding factors, including lifestyle, social status, and environmental stressors, which significantly reduces bias in causal deductions regarding the relationship between serum mineral content and chronic inflammatory skin diseases. Presently, the link between serum mineral levels and the prevalence of chronic inflammatory skin conditions including PS, AD, and AV remains controversial in clinical research, presumably owing to confounding variables (11). Furthermore, no large-scale, comprehensive assessments employing MR have been conducted. Consequently, the present investigation adopted a two-sample analysis strategy to highlight the critical importance of the correlation between serum mineral elements and chronic inflammatory skin disorders.

## 2 Materials and methods

### 2.1 Illustration of the three core assumptions in Mendelian randomization (MR) analysis

**Figure 1:**
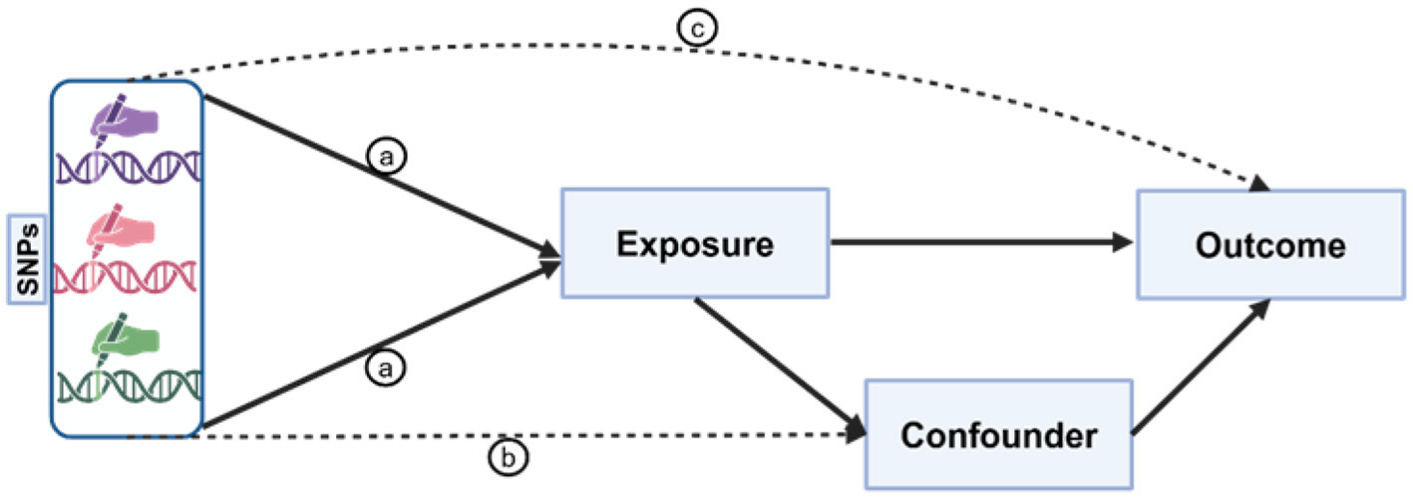
Illustration of the three core assumptions of Mendelian randomization (MR). Pathway “a” is the assumption of association, indicating a direct and stable relationship between genetic variants and exposure factors; pathway “b” is the assumption of independence, implying that genetic variants exclude all potential confounding factors that may affect the outcome; pathway “c” is the assumption of exclusivity, suggesting that genetic variants can only influence the outcome through their impact on exposure factors. These assumptions form the basis of MR analysis, ensuring the accuracy and reliability of causal inference.

This study adhered to the three essential criteria for MR: the IVs demonstrated significant associations with the exposure factors (*p* < 5 × 10^−8^), were free from confounding influences, and maintained a direct causal relationship without inverse causation (*p* > 5 × 10^−5^; Figure 1). Thus, a dual-sample MR analysis was conducted to elucidate the causal relationships among five serum minerals (Fe, Cu, Zn, Ca, and Se), iron metabolism (TIBC, TSAT, ferritin), and three chronic inflammatory skin diseases (PS, AD, and AV), followed by rigorous validation of SNP-outcome associations. Our investigation revealed a significant positive correlation between transferrin saturation (TSAT) and PS onset. Furthermore, elevated serum concentrations of Zn and Se were found to effectively mitigate the risk of AD. Conversely, the concentrations of the five minerals showed no significant association with AV.

### 2.2 Exposure measures

Exposure data for essential minerals (Fe, Cu, Zn, Ca, and Se) were obtained from the NHGRI-EBI genome-wide association study (GWAS; https://www.ebi.ac.uk/gwas). To ensure precision in selecting genetic instrumental variables for iron metabolism, we examined the total iron-binding capacity (TIBC), TSAT, and ferritin genetic variants. Initially, SNPs were chosen based on prior association studies and GWAS data, utilizing a stringent *p*-value threshold (typically 5 × 10^−8^), and Linkage Disequilibrium (LD) clumping was performed to confirm the independence of the SNPs. These selections were based on their well-established associations with iron indices in previous GWAS, thereby ensuring the relevance and specificity of these variables as proxies for iron exposure (17). The datasets for TIBC (*n* = 135,430), TSAT (*n* = 131,471), ferritin (*n* = 246,139), and Fe (*n* = 163,511) were derived from individuals of European ancestry (18). Genetic variants associated with serum Cu, Zn, and Se levels were extracted from the Queensland Institute of Medical Research Twin and Family Study (*n* = 2,603) (19). Serum Ca-related genetic variants were identified from the GWAS results involving 17 diverse populations (*n* = 39,400) (20) (Tables 1, 2). In our Mendelian randomization study, we mandated that all genetic instruments exhibit an F-statistic exceeding 10, thereby ensuring their robustness and substantially reducing the potential for bias. Furthermore, to guarantee the independence of the selected genetic variants across the eight exposure factors, we configured the linkage disequilibrium (LD) analysis with a window size of 10,000 kb and an *r*^2^ threshold below 0.001.

**Table 1:** Mineral information.

**Exposure**	**nSNPs**	**Sample size**	**Ancestry**
Fe	13	163,511	Europe
Cu	2	2,603	Europe
Zn	2	2,603	Europe
Se	2	2,603	Europe
Ca	6	39,400	Mix + Europe

**Table 2:** Iron metabolism information.

**Exposure**	**nSNPs**	**Sample size**	**Ancestry**
TSAT	13	163,511	Europe
TIBC	14	135,430	Europe
Ferritin	32	246,139	Europe

### 2.3 Result measurement

Three chronic inflammatory skin conditions, PS, AD, and AV were the focus of this study. PS data were procured from the Finnish database, encompassing a cohort of 6,408 afflicted individuals and 387,564 controls. Data pertaining to AD were similarly obtained from the Finnish dataset, featuring a robust sample size of 15,208 cases juxtaposed with 367,046 controls (21, 22). The dataset for AV was curated from the Finnish database, comprising 3,245 identified cases, and compared with a large cohort of 394,105 controls (23, 24) (Table 3).

**Table 3:** Data for the three chronic inflammatory skin diseases.

**Outcome**	**Total**	**Sample size**		
		**Cases**	**Controls**	**Ancestry**
PS	393,972	6,408	387,564	Europe
AD	382,254	15,208	367,046	Europe
AV	397,350	3,245	394,105	Europe

### 2.4 Statistical analysis of MR

Two-sample MR analyses of mineral-related measurements and chronic inflammatory skin diseases were performed using the “TwoSampleMR” package in the software R (version 4.1.2). We employed the inverse variance weighted (IVW) method to rigorously evaluate the comprehensive causal impact of specific exposure factors on the outcome variable. Odds ratios (ORs) and their 95% confidence intervals (CIs) were used to precisely quantify the magnitude and direction of the causal association; OR values significantly departing from 1 (95% CI not including 1) usually signify a noteworthy causal connection. To ensure the robustness of our findings and the reliability of the IVW assumptions, we also used the maximum likelihood estimation (MLE) method. Moreover, the MR-Egger method was employed to scrutinize the consistency of the IVW results and identify potential horizontal pleiotropy bias, where a *p*-value for the intercept exceeding 0.05 commonly suggests the absence of significant horizontal pleiotropy.

To scrutinize the heterogeneity among the instrumental variables, a *Q*-test was conducted, wherein higher *p*-values suggested insignificant heterogeneity, thereby implying the uniformity of instrumental variable estimates. Sensitivity analyses were conducted using weighted median, simple median, and leave-one-out methods to scrutinize the robustness of the MR analysis outcomes. These sensitivity analyses serve to safeguard against undue influence from individual instrumental variable outliers or potential biases, thus enhancing confidence in the IVW analysis results.

## 3 Result

### 3.1 Causality between minerals and psoriasis

**Figure 2:**
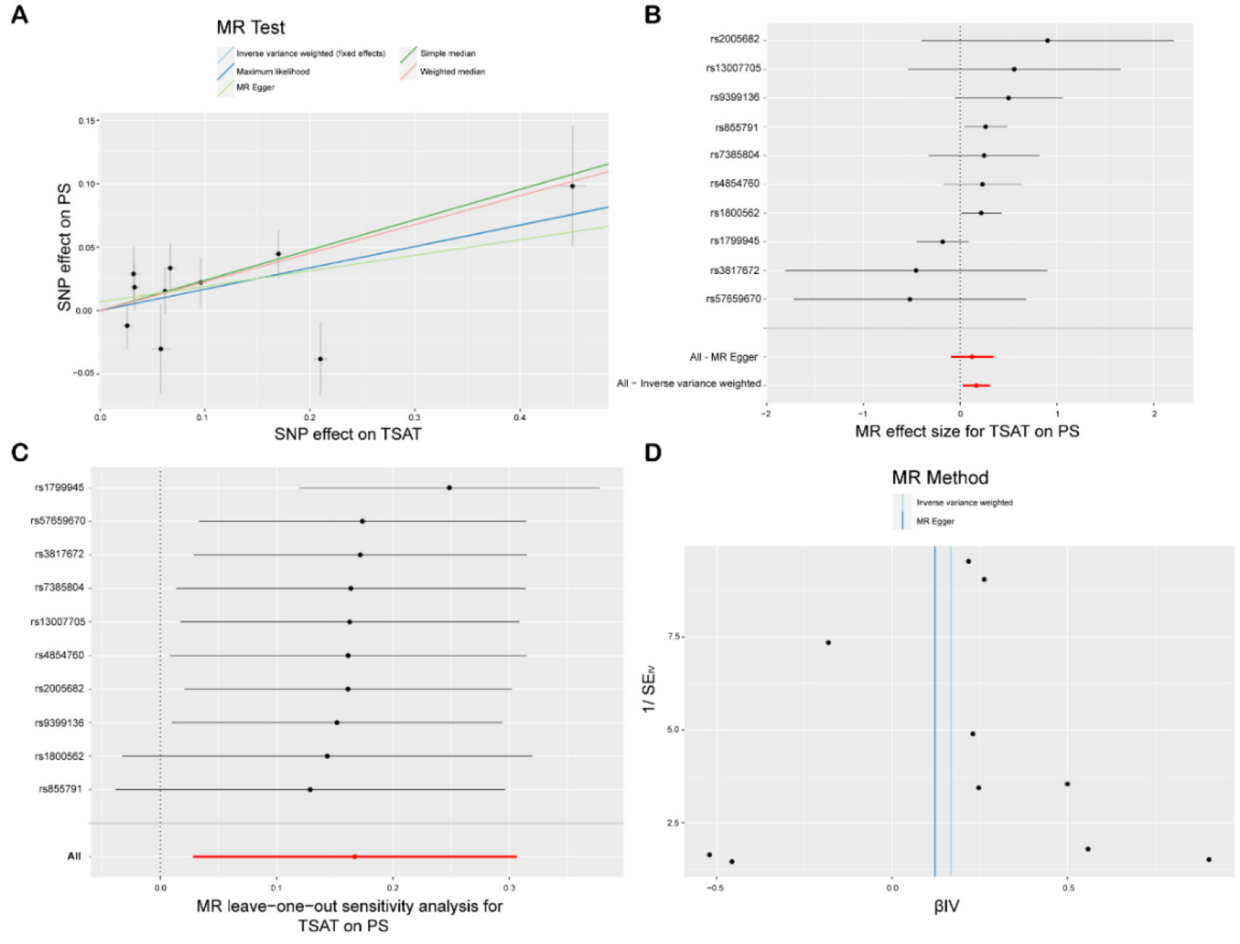
MR study of TSAT and PS. **(A)** Scatterplot: the *X*-axis represents standard deviation units, signifying the magnitude of SNP effects on transferrin saturation (TSAT), and the *Y*-axis represents the logarithmic odds ratio, elucidating the extent of SNP effects on psoriasis (PS). The slope of the line corresponds to causal estimates obtained using various methodologies. **(B)** Forest plot: individual SNP IV results are depicted by black dots, with red dots denoting the IVW results encompassing all SNPs, while horizontal lines represent the 95% confidence interval. **(C)** Leave-one-out sensitivity analysis: SNPs positioned far from 0 in the scatterplot imply that when the individual SNP is employed as an instrumental variable, its causal estimate for the outcome variable diverges significantly from zero, thereby indicating a potentially substantial impact of the SNP on the outcome variable. **(D)** Funnel plot: The inverse of the standard error of the causal estimate is estimated utilizing each SNP as an instrument, and the results are presented.

**Figure 3:**
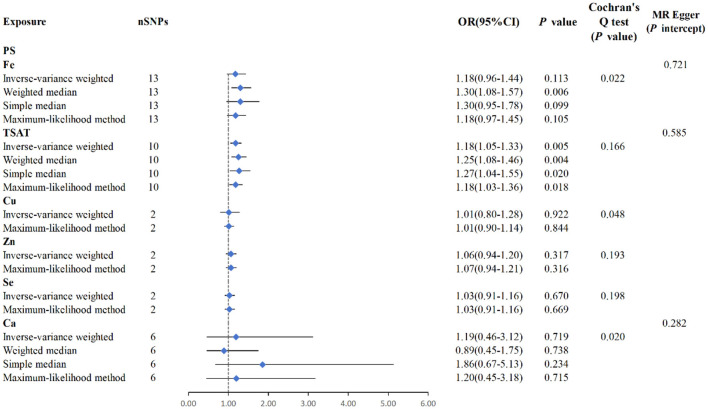
MR analysis between six exposures (Fe, TSAT, Cu, Zn, Se, Ca) and outcomes (PS). Three methods: maximum likelihood, weighted median, simple median, and IVW, Cochran's Q test, and MR-Egger method.

The IVW analysis revealed a significant association between TSAT, a critical marker for assessing blood iron levels, and PS [*p* = 0.004, odds ratio (OR) 95% confidence interval (CI) = 1.18 (1.05–1.32)]. A CI >1 suggests a positive causal relationship between increased TSAT levels and heightened PS risk; for each unit increase in TSAT, the average PS risk increased by 18%. Cochran's *Q* test and MR-Egger regression analysis were used to examine the single-nucleotide polymorphism (SNP) heterogeneity and horizontal pleiotropy, and Cochran's *Q* test (*p* = 0.16) indicated no notable heterogeneity in the effects on PS risk among the utilized genetic instruments, implying consistent impacts of all TSAT-related genetic variations on PS and bolstering the reliability of IVW outcomes. The MR-Egger analysis (intercept = 0.58, *p* = 0.26) revealed a lack of statistically significant deviation from 0 for the intercept term, indicating minimal horizontal pleiotropy in the analysis. Moreover, the findings from the weighted median (WM, *p* = 0.003), simple median (SM, *p* = 0.019), and maximum likelihood (MLM, *p* = 0.018) methods all yielded *p*-values below 0.05, reinforcing the reliability and robustness of our findings. Additionally, leveraging the “single SNP” and “leave-one-out” techniques, we identified rs1799945 as a significant mediator of the relationship between TSAT and PS (Figures 2, 3).

MR analysis using the IVW results revealed no genetic causality between serum mineral elements such as Fe [*p* = 0.113, OR 95% CI = 1.18 (0.96–1.44)], Cu [*p* = 0.922, OR 95% CI = 1.01 (0.80–1.28)], Zn [*p* = 0.317, OR 95% CI = 1.06 (0.94–1.20)], Se [*p* = 0.670, OR 95% CI = 1.03 (0.91–1.16)], or Ca [*p* = 0.719, OR 95% CI = 11.19 (0.46–3.12)] and PS (Figure 3). Furthermore, in the MR analysis, there was no causal relationship between iron-metabolism-related variables, such as TIBC and ferritin, and the onset of PS (Supplementary Figure S1).

### 3.2 Causality between minerals and atopic dermatitis

**Figure 4:**
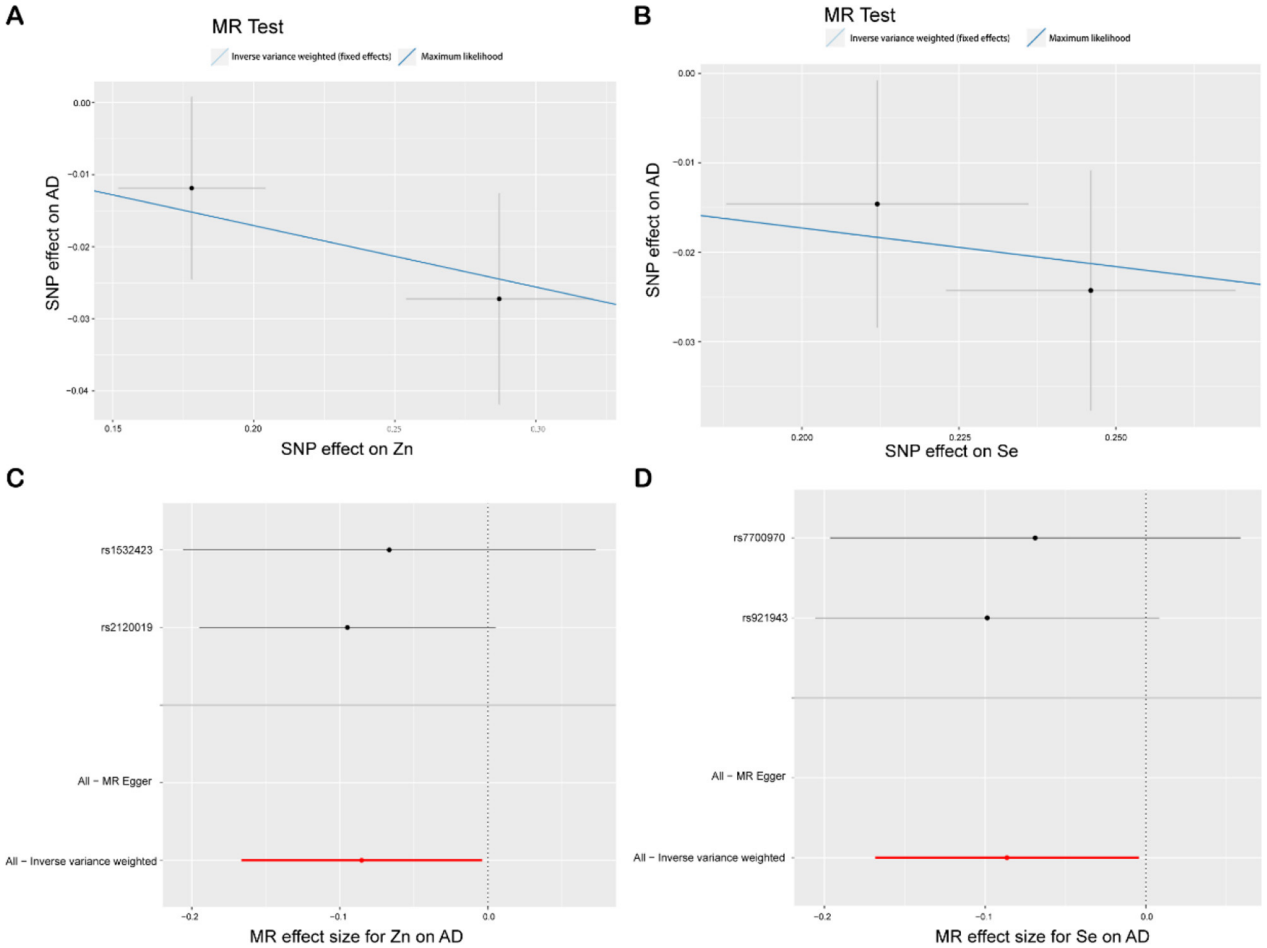
MR study of Zn, Se, and AD. **(A)** Scatterplot: The *X*-axis represents standard deviation units, signifying the magnitude of SNP effects on Zn, and the *Y*-axis represents the logarithmic odds ratio, elucidating the extent of SNP effects on AD. The slope of the line corresponds to causal estimates obtained using various methodologies. **(B)** Scatterplot: The *X*-axis represents standard deviation units, signifying the magnitude of SNP effects on Se, and the *Y*-axis portrays the logarithmic odds ratio, elucidating the extent of SNP effects on AD. The slope of the line corresponds to causal estimates obtained using various methodologies. **(C, D)** Forest plot: Individual SNP IV results are depicted by black dots, with red dots denoting the IVW results encompassing all SNPs, and horizontal lines represent the 95% confidence interval.

**Figure 5:**
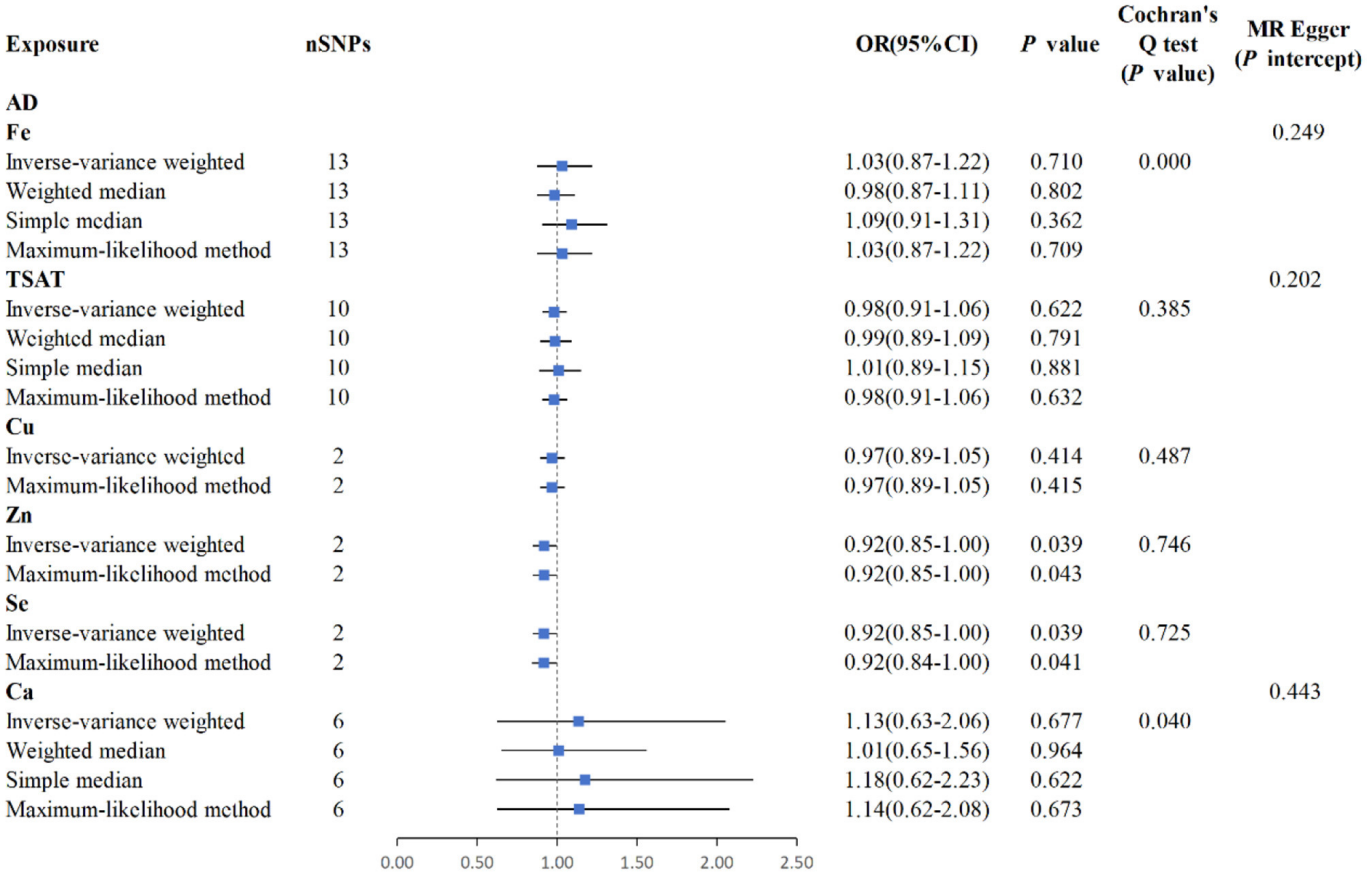
MR analysis for the six exposures (Fe, TSAT, Cu, Zn, Se, Ca) and outcomes (AD). Three methods: maximum likelihood, weighted median, simple median, and IVW; Cochran's *Q* test; MR-Egger.

The IVW analysis reveals a correlation between blood Zn and AD [*p* = 0.039, OR 95% CI = 0.92 (0.85–1.00)], with a *p*-value close to the conventional threshold of significance (0.05), suggesting statistical significance, despite being limited by the small number of SNPs. The OR of 0.92, with a 95% confidence interval ranging from 0.85 to 1.00, implies an average 8% reduction in AD risk per unit increase in Zn intake. Furthermore, the MLM analysis yields a *p*-value of 0.043, supporting the association between Zn intake and AD. Similarly, the IVW results for blood Se and AD reveal a negative correlation [*p* = 0.039, OR 95% CI = 0.92 (0.84–1.00)], with the MLM analysis producing a *p*-value of 0.041, thus corroborating the association between Se intake and AD. Conversely, Fe, Cu, Ca, and iron metabolism (TIBC, TSAT, ferritin) do not show a correlation with AD based on the IVW analysis. Overall, the analysis suggested a slight statistical correlation between Zn and Se intake and AD, possibly due to the limited number of SNPs (Figures 4, 5, Supplementary Figure S1).

### 3.3 Causality between minerals and acne vulgaris

**Figure 6:**
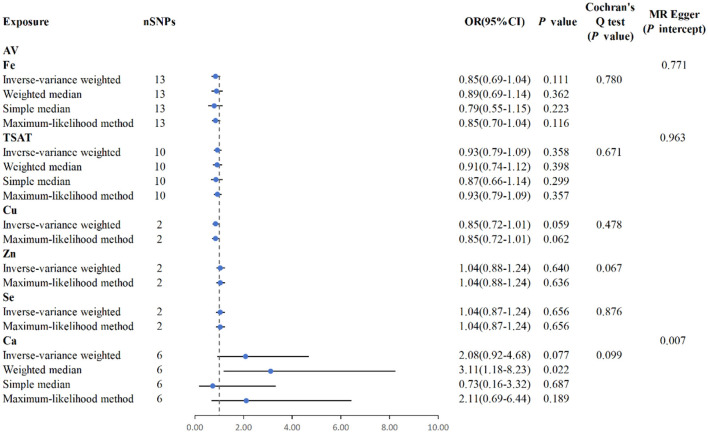
MR analysis for the six exposures (Fe, TSAT, Cu, Zn, Se, Ca) and outcomes (AV). Three methods: maximum likelihood, weighted median, simple median, and IVW; Cochran's *Q* test; MR-Egger.

MR analysis using the IVW results reveal no genetic causality between serum mineral elements such as Fe [*p* = 0.111, OR 95% confidence interval (CI) = 0.85 (0.69–1.04)], Cu [*p* = 0.059, OR 95% CI = 0.85 (0.72–1.01)], Zn [*p* = 0.640, OR 95% CI = 1.04 (0.88–1.24)], Se [*p* = 0.656, OR 95% CI = 1.04 (0.87–1.24)], and Ca [*p* = 0.077, OR 95% CI = 2.08 (0.92–4.68)] and AV (Figure 6). Furthermore, in the MR analysis, no causal relationship can be observed between iron-metabolism-related variables such as TIBC and ferritin, and the onset of AV (Supplementary Figure S1).

## 4 Discussion

The intake of mineral elements plays a pivotal role in maintaining the internal milieu and physiological functionality of humans. Deficiencies in mineral intake or disruptions in metabolic processes, whether hereditary or acquired, are frequently associated with dermatological conditions (25). For example, inadequate Zn levels can precipitate perioral or acrodermatitis characterized by pustular eruptions (26). Similarly, Cu deficiency may result in depigmentation of the skin (25). In recent years, research focusing on the influence of mineral elements on chronic inflammatory skin diseases such as PS, AV, and AD has surged; however, this surge has also produced some contentious findings. For example, Wacewicz et al. (27) reported that a Se deficiency may trigger inflammatory skin conditions such as PS, while research conducted by Toossi et al. (28) indicated that there was no significant difference in Se levels between patients with PS and the control group. This disparity in outcomes may be ascribed to a multitude of confounding variables linked to mineral consumption, including dietary patterns and educational attainment. The homeostasis of serum iron levels plays a crucial role in regulating immune cell function, with iron deficiency in the serum promoting Th2 cell survival, immunoglobulin class switching, and triggering mast cell degranulation. The heightened immune response in patients with atopic diseases increases their susceptibility to autoimmune conditions, with iron homeostasis imbalances potentially rooted in genetic factors. Maintaining optimal iron levels during pregnancy is associated with a reduced risk of atopic dermatitis and asthma in offspring. Moreover, patients suffering from chronic inflammatory skin diseases often present with hypotension and peripheral edema, which are driven by enhanced vasodilation and increased vascular permeability. Research has shown that anemia affects 27% of psoriasis patients, primarily attributed to folate and iron deficiencies (29, 30). Ferritin, serum iron (Fe), total iron-binding capacity (TIBC), and transferrin saturation (TSAT) are critical biomarkers for evaluating iron metabolism and storage in the body. TIBC denotes the total capacity of transferrin in the bloodstream to bind iron, while TSAT reflects the ratio of serum iron to TIBC and is widely employed to evaluate transferrin's iron-binding capacity. TSAT indicates the percentage of iron bound to transferrin in circulation. Both abnormally high and low TSAT levels suggest a disruption in the body's iron homeostasis (31). The statistical technique of MR is employed to estimate the causal effects of particular exposures on outcomes, effectively surmounting the common confounding factors and issues of reverse causality prevalent in observational studies (16). To date, no large-scale MR-based causal analyses of mineral element intake and chronic inflammatory skin diseases have been conducted. Thus, the present investigation delved deeply into the causal relationships among five mineral elements (Fe, Cu, Zn, Se, and Ca), iron metabolism (TSAT, TIBC, and ferritin), and three prevalent chronic inflammatory skin conditions (PS, AD, and AV), offering a powerful approach for prioritizing intervention targets in clinical trials and fostering causal inferences in epidemiological and biomedical research.

PS is a chronic inflammatory skin disorder that affects ~2% of the global population (32). Chen et al. (11) demonstrated the pivotal role of trace elements in PS and conducted relevant meta-analyses; however, causal studies regarding the relationship between trace elements and PS, which controls the confounding factors, remain unreported. Iron plays a pivotal role in wound healing and the prevention of inflammation in the skin (33). It has been shown that in severe cases of PS, the ferritin-to-iron ratio is elevated, indicating an imbalance in iron homeostasis (34). Conversely, another clinical analysis of PS revealed a decrease in serum iron concentration in patients with PS (35). In a meta-analysis conducted by Chen et al. (11), no significant correlation was observed between serum Fe levels and PS. In the current MR analysis, no correlation can be found between serum Fe and PS, consistent with the findings of Chen et al. (11). However, we identified a positive correlation between TSAT and PS [*p* = 0.004, OR 95% CI = 1.18 (1.05–1.32)], with each unit increase in TSAT being associated with an average 18% increase in PS risk. TSAT represents the ratio of serum iron to total iron-binding capacity and serves as an indicator of iron-binding status. Several clinical studies have reported the co-occurrence of hereditary hemochromatosis and PS (36, 37). Thus, this study demonstrates a potential association between imbalanced iron homeostasis and the onset of PS, indirectly supporting a genetic correlation between the two. Furthermore, the meta-analysis by Chen et al. demonstrated that serum Cu and tissue Zn levels in patients with PS were significantly higher than those in the control group. However, evidence from MR studies based on the current European population indicates no significant genetic correlations between Cu, Zn, Se, Ca, and PS.

AD is a chronic inflammatory skin condition that affects ~3% of the global population. Its onset involves genetic, immune, and environmental factors, making its pathogenesis complex (38, 39). Vaughn et al. (40) systematically assessed the relationship between micronutrients, including vitamins C, E, and D, Zn, Cu, Fe, Se, Mg, and strontium (Sr), and AD, underscoring the potential exacerbation of AD due to deficiencies in Se and Zn. However, to date, no MR analyses have been conducted on mineral elements and AD. Based on our MR analysis conducted on the European ancestry level of the Finnish database, our findings revealed a correlation between Zn and Se levels and AD, with IVW *p*-values < 0.05. Our findings suggest a negative genetic correlation between Zn/Se and AD, indicating that an increase in serum Zn and Se levels may decrease the risk of AD. However, the OR 95% CIs for these two exposure factors were 0.92 (0.85–1.00) and 0.92 (0.84–1.00), respectively; these intervals contain 1.00, suggesting a relatively modest effect size for both exposure factors and a lack of robust causal relationships. We attribute this to the limited number of SNPs in the exposure factors, which affects the stability and reliability of the results. In this MR analysis, no causal relationship was found between serum Cu or Ca levels and AD. It is noteworthy that some researchers have found an increased risk of iron deficiency anemia in patients with atopic dermatitis (AD); however, there has been no causal investigation into whether iron deficiency anemia elevates the risk of developing AD (41). Additionally, the authors conducted MR analyses of the exposure factors related to iron metabolism (TSAT, ferritin, and TIBC) and AD, which yielded negative results. This contradicts the findings of Fortes et al. (42) whose study suggested an 80% reduction in the likelihood of offspring developing atopic dermatitis when maternal supplementation of serum iron and folic acid was administered. Therefore, we hypothesized that the reduction in the incidence of AD in the offspring could be primarily attributed to folic acid.

AV is a chronic inflammatory skin condition that affects ~15% of the global population. The onset of acne is often influenced by external factors such as improper skin care, air pollution, or diet (43). Because of the antioxidative and anti-inflammatory properties of Zn and Se (9, 44) research investigating their association with AV is expanding. Lv et al. (45) conducted a meta-analysis on the association between serum Se levels and AV, revealing a lower concentration of Se in AV patients, Ozuguz et al. (46) reported lower serum Zn concentrations in individuals with AV. In the study by Leyden (47), lower serum Fe levels were observed in patients with severe AV. However, to date, no specific studies have been conducted on the causal relationships between mineral elements and AV. In this MR analysis, focusing on European ancestry, no causal relationship was found between serum Fe and iron metabolism, or Cu, Zn, Se, and Ca levels and the onset of AV.

Current research indicates that mineral intake is influenced by various environmental and inherent factors; the occurrence and development of chronic inflammatory skin diseases are also influenced by immune, genetic, and psychological stressors. On the one hand, our study's strength lies in the dual-sample MR design, which minimizes confounding factors. Additionally, we utilized GWAS databases, with all populations restricted to European ancestry, reducing ethnic stratification bias. On the other hand, the limitation of this study is that only the Finnish database is utilized, potentially constraining the applicability and generalizability of our findings, as the studied population may have specific genetic backgrounds and levels of environmental exposure that differ from those of people of other ethnicities and geographic locations. The limited number of SNPs for Zn and Se (only two) may have affected the analytical statistical power and robustness of the causal inference regarding the association between these minerals and chronic inflammatory skin diseases. A smaller number of SNPs may lead to increased uncertainty in the estimates, thus affecting the interpretation of the results. Future research should consider using more diverse databases and increasing the number of SNPs in the MR analysis to strengthen the causal inferences of the relationship between mineral elements and chronic inflammatory skin diseases, thereby enhancing the generalizability of the study results.

## 5 Conclusion

Our investigation revealed an association between TSAT levels and genetic susceptibility to PS, underscoring the pivotal role of iron homeostasis disruption in the pathogenesis of PS. Additionally, our findings indicate that dietary consumption of Zn and Se may confer a protective effect against the development of AD; further empirical substantiation and validation of this point are needed. Furthermore, our analysis demonstrated the absence of a causal link between serum mineral levels and iron metabolism in relation to the etiology of AV.

## Data Availability

Publicly available datasets were analyzed in this study. Data for this submission are available in the following dataset repository: Psoriasis https://storage.googleapis.com/finngen-public-data-r10/summary_stats/finngen_R10_L12_PSORI_VULG.gz. Atopic dermatitis https://storage.googleapis.com/finngen-public-data-r10/summary_stats/finngen_R10_L12_ATOPIC.gz. Acne vulgaris https://storage.googleapis.com/finngen-public-data-r10/summary_stats/finngen_R10_L12_ACNE.gz.

## References

[1] Griffiths CEM Armstrong AW Gudjonsson JE Barker JNWN. Psoriasis. Lancet. (2021) 397:1301–15. 10.1016/S0140-6736(20)32549-6 33812489

[2] Sroka-Tomaszewska J Trzeciak M. Molecular mechanisms of atopic dermatitis pathogenesis. Int J Mol Sci. (2021) 22:4130 10.3390/ijms22084130 33923629 PMC8074061

[3] Vasam M Korutla S Bohara RA. Acne vulgaris: a review of the pathophysiology, treatment, and recent nanotechnology based advances. Biochem Biophys Rep. (2023) 36:101578. 10.1016/j.bbrep.2023.101578 38076662 PMC10709101

[4] Fan Y Mao R Yang J. NF-κB and STAT3 signaling pathways collaboratively link inflammation to cancer. Protein Cell. (2013) 4:176–85. 10.1007/s13238-013-2084-3 23483479 PMC4875500

[5] Hensler SMM. Inflammation and skin cancer: old pals telling new stories. Cancer J. (2013) 19:517–24. 10.1097/PPO.0000000000000010 24270351

[6] Pouplard C Brenaut E Horreau C Barnetche T Misery L Richard MA. Risk of cancer in psoriasis: a systematic review and meta-analysis of epidemiological studies. J Eur Acad Dermatol Venereol. (2013) 27:36–46. 10.1111/jdv.12165 23845151

[7] Zhu Y Wang H He J Yang L Zhou X Li Z. Atopic dermatitis and skin cancer risk: a systematic review. Dermatol Ther. (2022) 12:1167–79. 10.1007/s13555-022-00720-2 35430723 PMC9110609

[8] Ogawa Y Kawamura T Shimada S. Zinc and skin biology. Arch Biochem Biophys. (2016) 611:113–9. 10.1016/j.abb.2016.06.003 27288087

[9] Roman M Jitaru P Barbante C. Selenium biochemistry and its role for human health. Metallomics. (2014) 6:25–54. 10.1039/C3MT00185G 24185753

[10] Leveque N Robin S Muret P Mac-Mary S Makki S Berthelot A. *In vivo* assessment of iron and ascorbic acid in psoriatic dermis. Acta Derm Venereol. (2004) 84:2–5. 10.1080/00015550310014717 15040469

[11] Chen W Zhou X Zhu W. Trace elements homeostatic imbalance in psoriasis: a meta-analysis. Biol Trace Elem Res. (2019) 191:313–22. 10.1007/s12011-018-1626-1 30648223

[12] Cheng WW Zhu Q Zhang HY. Mineral nutrition and the risk of chronic diseases: a Mendelian randomization study. Nutrients. (2019) 11:378. 10.3390/nu11020378 30759836 PMC6412267

[13] Naji HH Al-Azawi RSA Ibrahim NJ Kzar HH. Investigation of the role of Zn/Cu Index and its correlation with physiological activity of SOD 1 and GRx in males with acne vulgaris. Arch Razi Inst. (2022) 77:623–8. 10.22092/ARI.2021.356857.1928 36284950 PMC9548251

[14] Boehm FJ Zhou X. Statistical methods for Mendelian randomization in genome-wide association studies: a review. Comput Struct Biotechnol J. (2022) 20:2338–51. 10.1016/j.csbj.2022.05.015 35615025 PMC9123217

[15] Zheng J Baird D Borges MC Bowden J Hemani G Haycock P. Recent developments in mendelian randomization studies. Curr Epidemiol Rep. (2017) 4:330–45. 10.1007/s40471-017-0128-6 29226067 PMC5711966

[16] Lamina C. Mendelian randomization: principles and its usage in Lp(a) research. Atherosclerosis. (2022) 349:36–41. 10.1016/j.atherosclerosis.2022.04.013 35606074

[17] Benyamin B Esko T Ried JS Radhakrishnan A Vermeulen SH Traglia M. Novel loci affecting iron homeostasis and their effects in individuals at risk for hemochromatosis. Nat Commun. (2014) 5:4926. 10.1038/ncomms5926 25352340 PMC4215164

[18] Bell S Rigas AS Magnusson MK Ferkingstad E Allara E Bjornsdottir G. A genome-wide meta-analysis yields 46 new loci associating with biomarkers of iron homeostasis. Commun Biol. (2021) 4:156. 10.1038/s42003-020-01575-z 33536631 PMC7859200

[19] Evans DM Zhu G Dy V Heath AC Madden PA Kemp JP. Genome-wide association study identifies loci affecting blood copper, selenium and zinc. Hum Mol Genet. (2013) 22:3998–4006. 10.1093/hmg/ddt239 23720494 PMC3766178

[20] O'Seaghdha CM Wu H Yang Q Kapur K Guessous I Zuber AM. Meta-analysis of genome-wide association studies identifies six new Loci for serum calcium concentrations. PLoS Genet. (2013) 9:e1003796. 10.1371/journal.pgen.1003796 24068962 PMC3778004

[21] Zhou W Cai J Li Z Lin Y. Association of atopic dermatitis with autoimmune diseases: a bidirectional and multivariable two-sample mendelian randomization study. Front Immunol. (2023) 14:1132719. 10.3389/fimmu.2023.1132719 37063839 PMC10098361

[22] Meisinger C Freuer D. Causal association between atopic dermatitis and inflammatory bowel disease: a 2-sample bidirectional Mendelian randomization study. Inflamm Bowel Dis. (2022) 28:1543–8. 10.1093/ibd/izab329 34964870

[23] Liu L Xue Y Chen Y Chen T Zhong J Shao X. Acne and risk of mental disorders: a two-sample Mendelian randomization study based on large genome-wide association data. Front Public Health. (2023) 11:1156522. 10.3389/fpubh.2023.1156522 37064666 PMC10102334

[24] Cao Q Guo J Chang S Huang Z Luo Q. Gut microbiota and acne: a Mendelian randomization study. Skin Res Technol. (2023) 29:e13473. 10.1111/srt.13473 37753688 PMC10507220

[25] DiBaise M Tarleton SM. Hair, nails, and skin: differentiating cutaneous manifestations of micronutrient deficiency. Nutr Clin Pract. (2019) 34:490–503. 10.1002/ncp.10321 31144371

[26] Galimberti F Mesinkovska NA. Skin findings associated with nutritional deficiencies. Cleve Clin J Med. (2016) 83:731–9. 10.3949/ccjm.83a.15061 27726828

[27] Wacewicz M Socha K Soroczynska J Niczyporuk M Aleksiejczuk P Ostrowska J. Concentration of selenium, zinc, copper, Cu/Zn ratio, total antioxidant status and c-reactive protein in the serum of patients with psoriasis treated by narrow-band ultraviolet B phototherapy: a case-control study. J Trace Elem Med Biol. (2017) 44:109–14. 10.1016/j.jtemb.2017.06.008 28965564

[28] Toossi P Sadat Amini SH Sadat Amini MS Partovi Kia M Enamzade R Kazeminejad A. Assessment of serum levels of osteopontin, selenium and prolactin in patients with psoriasis compared with healthy controls, and their association with psoriasis severity. Clin Exp Dermatol. (2015) 40:741–6. 10.1111/ced.12657 25991399

[29] Roth-Walter F. Iron-deficiency in atopic diseases: innate immune priming by allergens and siderophores. Front Allergy. (2022) 3:859922. 10.3389/falgy.2022.859922 35769558 PMC9234869

[30] Wu C Yu C Yang Y Jin H. Heart failure in erythrodermic psoriasis: a retrospective study of 225 patients. Front Cardiovasc Med. (2023) 10:1169474. 10.3389/fcvm.2023.1169474 37593148 PMC10427504

[31] Elsayed ME Sharif MU Stack AG. Transferrin saturation: a body iron biomarker. Adv Clin Chem. (2016) 75:71–97. 10.1016/bs.acc.2016.03.002 27346617

[32] Deng Y Chang C Lu Q. The inflammatory response in psoriasis: a comprehensive review. Clin Rev Allergy Immunol. (2016) 50:377–89. 10.1007/s12016-016-8535-x 27025861

[33] Wright JA Richards T Srai SK. The role of iron in the skin and cutaneous wound healing. Front Pharmacol. (2014) 5:156. 10.3389/fphar.2014.00156 25071575 PMC4091310

[34] Jishna P Dominic S. Acute phase reactants: relevance in dermatology. Indian Dermatol Online J. (2023) 14:1–8. 10.4103/idoj.idoj_174_21 36776186 PMC9910534

[35] Shahidi-Dadras M Namazi N Younespour S. Comparative analysis of serum copper, iron, ceruloplasmin, and transferrin levels in mild and severe psoriasis vulgaris in Iranian patients. Indian Dermatol Online J. (2017) 8:250–3. 10.4103/idoj.IDOJ_230_16 28761840 PMC5518575

[36] Ruan DDGY Lu T Yang X Zhu YB Yu QH Liao LS. Genetic diagnosis history and osteoarticular phenotype of a nontransfusion secondary hemochromatosis. World J Clin Cases. (2020) 8:5962–75. 10.12998/wjcc.v8.i23.5962 33344595 PMC7723718

[37] Godde A Muller-Ladner U. Therapy for secondary arthropathies: from psoriasis and Reiter's disease to hemochromatosis. Internist. (2006) 47:1263–8. 10.1007/s00108-006-1754-0 17102998

[38] Omata NTH Ito S Ohshima Y Yasutomi M Yamada A Jiang M. Increased oxidative stress in childhood atopic dermatitis. Life Sci. (2001) 69:223–8. 10.1016/S0024-3205(01)01124-9 11441912

[39] Mei-Yen Yong A Tay YK. Atopic dermatitis: racial and ethnic differences. Dermatol Clin. (2017) 35:395–402. 10.1016/j.det.2017.02.012 28577807

[40] Vaughn AR Foolad N Maarouf M Tran KA Shi VY. Micronutrients in atopic dermatitis: a systematic review. J Altern Complement Med. (2019) 25:567–77. 10.1089/acm.2018.0363 30912673

[41] Rhew K Brown JD Oh JM. Atopic disease and anemia in korean patients: cross-sectional study with propensity score analysis. Int J Environ Res Public Health. (2020) 17:1978. 10.3390/ijerph17061978 32197291 PMC7142528

[42] Fortes C Mastroeni S Mannooranparampil TJ Di Lallo D. Pre-natal folic acid and iron supplementation and atopic dermatitis in the first 6 years of life. Arch Dermatol Res. (2019) 311:361–7. 10.1007/s00403-019-01911-2 30923900

[43] Podgorska A Puscion-Jakubik A Markiewicz-Zukowska R Gromkowska-Kepka KJ Socha K. Acne vulgaris and intake of selected dietary nutrients-a summary of information. Healthcare. (2021) 9:668. 10.3390/healthcare9060668 34205209 PMC8226785

[44] Chasapis CT Ntoupa PA Spiliopoulou CA Stefanidou ME. Recent aspects of the effects of zinc on human health. Arch Toxicol. (2020) 94:1443–60. 10.1007/s00204-020-02702-9 32394086

[45] Lv J Ai P Lei S Zhou F Chen S Zhang Y. Selenium levels and skin diseases: systematic review and meta-analysis. J Trace Elem Med Biol. (2020) 62:126548. 10.1016/j.jtemb.2020.126548 32497930

[46] Ozuguz P Dogruk Kacar S Ekiz O Takci Z Balta I Kalkan G. Evaluation of serum vitamins A and E and zinc levels according to the severity of acne vulgaris. Cutan Ocul Toxicol. (2014) 33:99–102. 10.3109/15569527.2013.808656 23826827

[47] Leyden JJ. Low serum iron levels and moderate anemia in severe nodulocystic acne. Reversal with isotretinoin therapy. Arch Dermatol. (1985) 121:214–5. 10.1001/archderm.121.2.214 3156559

