# Supplementary material for: A Mendelian randomization analysis of inflammatory skin disease risk due to mineral deficiencies
Source: Front Nutr. 2024 Oct 14;11:1404117. doi: 10.3389/fnut.2024.1404117 (PMC11513277; doi:10.3389/fnut.2024.1404117)
Supplement: Supplementary file 1 [file Image_1.pdf]

	SNPs	P value	OR	95%CI (low)	95%CI (upper)	P interc	Qrs
PS							
TIBC-2021							
Inverse-v	14	0.205706516	0.926837233	0.823942673	1.042581339	NA	0.0764492
Inverse-v	14	0.109226105	0.926837233	0.844549588	1.017142472	NA	0.0764492
MR-Egger	14	0.305167854	0.922456764	0.790574926	1.076338817	0.921208	NA
Weightec	14	0.115574382	0.915134973	0.819433392	1.022013537	NA	NA
simple m	14	0.795715757	1.038696663	0.779217713	1.38458192	NA	NA
Maximum-l	14	0.204654139	0.92652677	0.823465177	1.042487138	NA	NA
Ferritin-2021							
Inverse-v	32	0.985653853	1.0023044	0.77989561	1.288139204	NA	0.0130725
Inverse-v	32	0.981587868	1.0023044	0.824331639	1.218701385	NA	0.0130725
MR-Egger	32	0.989170557	0.996419388	0.593589865	1.672622217	0.979575	NA
Weightec	32	0.213953287	0.818888884	0.597544103	1.122225123	NA	NA
simple m	32	0.56984531	0.909804097	0.656687611	1.260482885	NA	NA
Maximum-l	32	0.985395736	1.00236625	0.778251259	1.291020204	NA	NA
AD							
TIBC-2021							
Inverse-v	14	0.175982108	1.057302825	0.975324929	1.146171118	NA	0.0544337
Inverse-v	14	0.077932061	1.057302825	0.99378645	1.124878755	NA	0.0544337
MR-Egger	14	0.841505992	0.991149529	0.908441542	1.081387567	0.016903	NA
Weightec	14	0.29007555	1.039086044	0.967837476	1.115579666	NA	NA
simple m	14	0.125484637	1.218649555	0.946277962	1.569419132	NA	NA
Maximum-l	14	0.176416339	1.057376328	0.97521534	1.146459303	NA	NA
Ferritin-2021							
Inverse-v	32	0.329893726	0.920919487	0.780276311	1.086913301	NA	0.0154719
Inverse-v	32	0.214362229	0.920919487	0.808619834	1.048815111	NA	0.0154719
MR-Egger	32	0.604093888	0.913439447	0.648709649	1.286201963	0.957208	NA
Weightec	32	0.271344548	0.892111266	0.727901221	1.093366087	NA	NA
simple m	32	0.137091987	0.856305678	0.697927149	1.050624575	NA	NA
Maximum-l	32	0.32595161	0.919666223	0.778146008	1.086924501	NA	NA
AV							
TIBC-2021							
Inverse-v	14	0.703164036	0.961642121	0.786395215	1.175942518	NA	0.0039926
Inverse-v	14	0.559471419	0.961642121	0.843271577	1.09662841	NA	0.0039926
MR-Egger	14	0.487953963	0.91231732	0.703913694	1.182421792	0.513217	NA
Weightec	14	0.363753926	0.929982739	0.795138377	1.087694821	NA	NA
simple m	14	0.150351586	0.771792302	0.542243144	1.098517085	NA	NA
Maximum-l	14	0.701524711	0.961336991	0.785782004	1.176113485	NA	NA
Ferritin-2021							
Inverse-v	32	0.247029551	0.842373953	0.630046187	1.126256918	NA	0.3071518
Inverse-v	32	0.222516378	0.842373953	0.639456486	1.109682821	NA	0.3071518
MR-Egger	32	0.790165025	1.083495952	0.600202815	1.955944641	0.337244	NA
Weightec	32	0.282565214	0.808773067	0.549167231	1.191101429	NA	NA
simple m	32	0.26515341	0.799391051	0.539158562	1.185228423	NA	NA
Maximum-l	32	0.245797674	0.84121405	0.62819125	1.126473948	NA	NA

Figure S1: The Mendelian randomization (MR) results pertaining to exposure factors associated with iron metabolism (TIBC, Ferritin) and their impact on psoriasis (PS), atopic dermatitis (AD), and acne vulgaris (AV) encompass a range of statistical methods, including inverse variance weighted (IVW), MR-Egger, weighted median, simple median, maximum-likelihood method, as well as odds ratios (OR) and 95% confidence intervals (CI).
